# Children’s social connections, internet usage and mental health during the COVID-19 pandemic in the Global South: evidence from *Disrupting Harm*

**DOI:** 10.1007/s00127-026-03101-8

**Published:** 2026-08-04

**Authors:** Thomas E. Metherell, Sebastian Kurten, Sakshi Ghai, George B. Ploubidis, Darío Moreno-Agostino, Amy Orben

**Affiliations:** 1 Centre for Longitudinal Studies, Social Research Institute, University College London, London, UK; 2 MRC Cognition & Brain Sciences Unit, University of Cambridge, Cambridge, UK; 3 Department of Interdisciplinary Social Science, Utrecht University, Utrecht, NL Netherlands; 4 Oxford Internet Institute, University of Oxford, Oxford, UK; 5 Department of Psychological and Behavioural Science, London School of Economics and Political Science, London, UK; 6 ESRC Centre for Society and Mental Health, King’s College London, London, UK

**Keywords:** Adolescence, COVID-19, Cross-sectional, Global South, Internet use, Mental health

## Abstract

**Purpose:**

There is a notable lack of studies investigating the mental health associations of the COVID-19 pandemic in the Global South, particularly with regard to the use of digital technologies. We aimed to investigate the cross-sectional associations of digital technology use with mental health indicators during the pandemic in 12 countries in the Global South.

**Methods:**

We used data from the UNICEF Innocenti *Disrupting Harm* survey. The survey included a representative sample of 11,912 internet-using children aged 12–17 in Ethiopia, Kenya, Mozambique, Namibia, Tanzania, Uganda, Cambodia, Indonesia, Malaysia, Philippines, Thailand and Vietnam. We modelled the associations of social connection during lockdown and internet use during the pandemic with six different indicators of wellbeing in each country (excluding Tanzania, where there had been no lockdowns) and in the overall sample using robust linear and logistic regression. We controlled for a range of putative socioeconomic and demographic confounders and handled missing data using multiple imputation.

**Results:**

We did not find clear evidence for any general associations of social connection or internet use with mental health indicators during lockdown across countries. Rather, our results are complex and demonstrate that the relationship depends heavily on an individual’s context; not just on the country they are living in but also on their gender, urbanicity and other factors. Nonetheless, our results highlight key focus areas for further research.

**Conclusion:**

Extensive further research is needed to identify ways in which technology use, including internet use, might act as a risk or protective factor relevant to mental ill health among young people in the countries of the Global South.

**Supplementary Information:**

The online version contains supplementary material available at https://doi.org/10.1007/s00127-026-03101-8.

## Introduction

A large international body of scientific evidence has reported deleterious mental health associations with the COVID-19 pandemic. For example, cross-sectional evidence from four countries in the Global South suggests that the rates of symptoms of anxiety and depression mirrored COVID-19 mortality rates [1]. A cross-sectional study in Colombia also found reduced average life satisfaction during the pandemic compared with baseline levels [2]. Among children and adolescents, the incidences of depression and anxiety appeared to be elevated worldwide during the pandemic compared to the period before according to an umbrella review [3]. Many of the identified studies pointed to the isolating effect of COVID-19 lockdowns, as well as disruptions to schooling, as reasons for increases in the rates of psychological distress among young people during the pandemic.

The existing literature has a notable lack of studies investigating the wellbeing implications of the COVID-19 pandemic in the Global South, and this becomes more pronounced in certain regions, most notably Africa [3]. It has also, for example, been noted that there existed no model of excess suicidality in low- and middle-income countries during the pandemic, in spite of the vast majority of suicides occurring in these countries (as a product of their greater share of the global population) [4]. This lack of investigation presented an urgent need for evidence, one that has recently started to be met with a report finding minimal changes in global prevalence of youth suicide mortality in the early stages of the pandemic [5].

In the face of the potential harm to mental health from social isolation, it was hypothesised that adolescents turned to online activities and methods of communication to mitigate this isolation [6]. There is some evidence to suggest that access to digital technology during the height of the pandemic was associated with lesser increases in mental health symptoms [7], suggesting that access to technologies could have such a protective effect. However, at the same time there is some controversy over purported negative mental health consequences arising from heavy social media use, and by extension internet use in general. To date, there is a lack of consistent longitudinal evidence that such negative consequences exist at the population level, and there is some evidence that online social connection through social media may be associated with higher well-being as well as higher ‘ill-being’ [8]. Furthermore, there is a distinct lack of existing studies investigating digital wellbeing outside the Global North [9]. Given the increasing importance of digital communication during the COVID-19 pandemic, in Global South countries as much as in the Global North, this is an important shortcoming of the existing literature. Currently there do not exist widely accepted theoretical frameworks that can guide investigation of adolescent technology use during the pandemic, especially in the Global South, yet the need for evidence in this area persists.

This study analyses data from *Disrupting Harm*, a survey of nearly 12,000 internet-using adolescents aged 12–17 in 12 countries in Africa and Asia conducted in 2020–2021. The survey was conducted as soon as possible after lockdowns were lifted in the countries involved. The primary aim of the survey was to investigate online child sexual exploitation and abuse in these countries, but the focus of this particular study is the relationship between pandemic-related social isolation and mental health, modulated or not by access to digitally mediated communication. Therefore, we cross-sectionally relate the questionnaire’s measures of social connection and internet use during the pandemic with indicators of mental health. In so doing, we aim to determine whether becoming disconnected from family and friends, and increased internet use, might variously be plausible risk or protective factors for adolescent mental health during the pandemic.

## Methods

### Sample and study characteristics

The *Disrupting Harm* survey was designed by UNICEF Innocenti – Global Office of Research and Foresight and conducted in collaboration with IPSOS between November 2020 and November 2021. Data were collected in 6 countries in sub-Saharan Africa (Ethiopia, Kenya, Mozambique, Namibia, Tanzania and Uganda) and 6 countries in southeast Asia (Cambodia, Indonesia, Malaysia, Philippines, Thailand and Vietnam). The target population comprised 12–17-year-old children living in private households who had used the internet in the three months before interview. The survey sample can be rendered representative of that target population through application of the supplied sampling weights. Reports on these data are available online [10] and this study is a secondary analysis of the data contained within those reports.

**Fig. 1:**
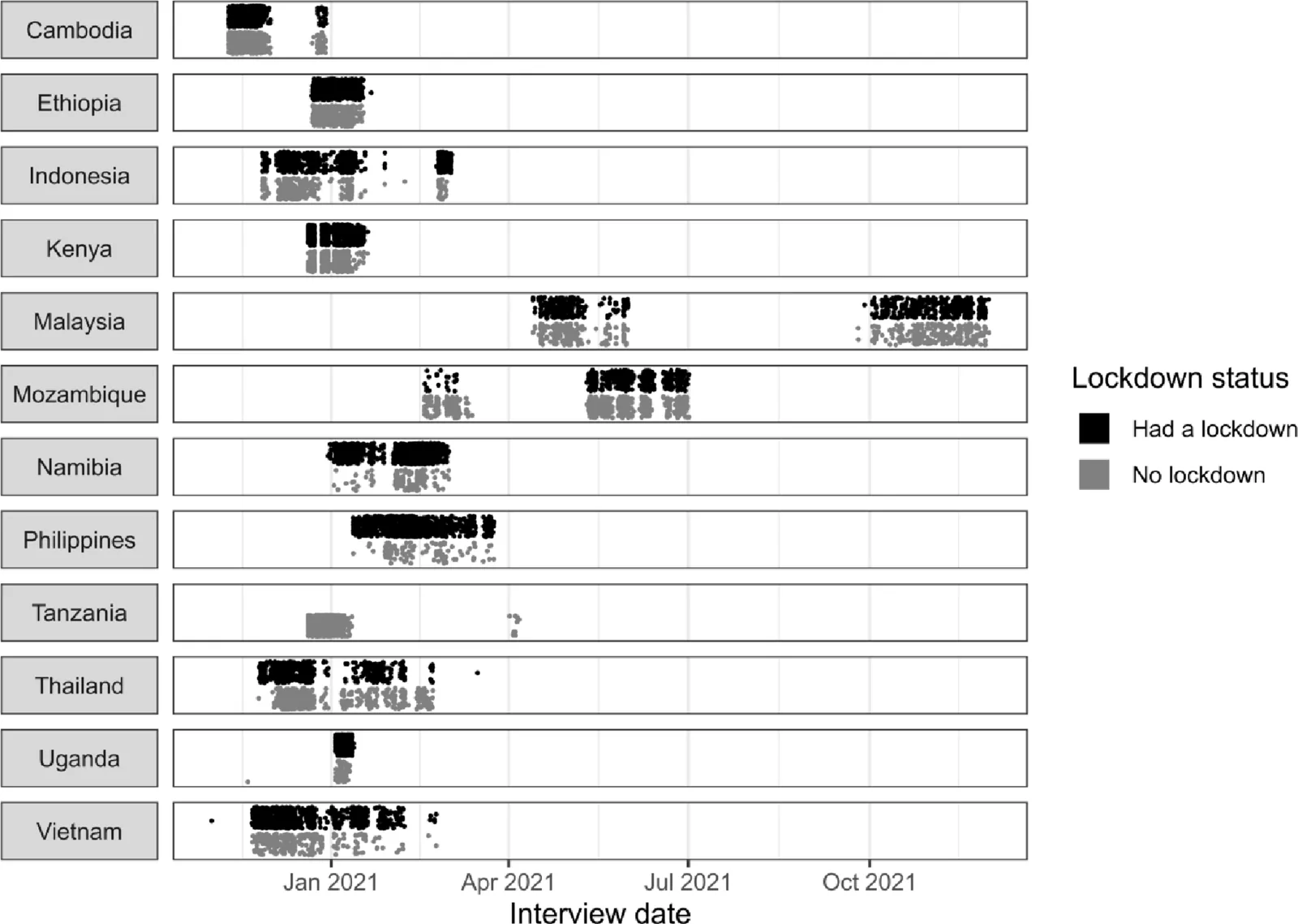
Plot showing interview dates for each participant in each country, alongside whether or not they reported having experienced a lockdown in their community

Participants were recruited via a three-stage clustered random sampling method. Primary sampling units (PSUs) were first drawn at random from the applicable sampling frame (after being stratified by region in some countries). Addresses within PSUs were then selected by interviewers using random walk procedures. Finally, individuals within successfully contacted households were selected at random (subject to the requirement that they had used the internet in the three months prior to contact) to be interviewed. Data collection within each country occurred within one or two short (around 1–3-month) windows, and there were no obvious systematic differences in whether children had experienced COVID-19 lockdowns because of differences in interview dates (see Fig. 1). The sampling frames in some countries were constrained by security considerations and the geographical isolation of certain regions, but each sampling frame had a minimum of 94% population coverage except for those of Indonesia (76%) and Ethiopia (82%). Participants with valid responses recorded to 70% or less of the survey items (257 participants in total) were systematically excluded from analysis. With these excluded, 11,912 children were included in the final sample, with at least 950 and at most 1,016 from each country.

Sampling weights were calculated by Ipsos to reflect the differences in probability of selection across participants. The weights were calculated in three stages. Firstly, design (inverse probability) weights accounted for the selection of PSUs, the selection of addresses within those PSUs and the selection of one individual to be interviewed at each address. Secondly, non-response weights adjusted for the bias caused by selective non-response. Finally, post-stratification weights calibrated the sample to the external population profile, based on United Nations Development Programme figures. The result is that the weighted sample can be considered to approximate a random sample of the internet-using population aged 12–17 in each country.

### Data analysis

We analysed six outcome measures related to the mental health and wellbeing of participants, which are outlined in Table 1. Multi-item measures were reduced to a single measure (derived factor score) by confirmatory factor analysis (CFA) with full information maximum likelihood estimation to handle missing responses. We then assessed each of the multi-item measures for metric invariance, i.e. whether the construct being measured by each scale was the same across countries. We did this by constraining the factor loadings of the CFA models to be equal across countries and comparing the fits of those models with those of the unconstrained models. As reasonable evidence for metric invariance, we accepted a change in comparative fit index (ΔCFI) of − 0.02 or smaller on constraint [11]. The applicable ΔCFI values are also shown in Table 1.

**Table 1:** Mental health and wellbeing measures analysed in this study

Outcome measure	What it measures	Number of items	ΔCFI	Final variable type, regression method
Cantril life satisfaction ladder	Life satisfaction	1	N/A	Continuous, robust linear
Modified Children’s Worlds Psychological Well-Being Scale (CW-PWBS)	Psychological wellbeing	6	−0.015	Continuous, robust linear
UNICEF Youth Empowerment Project anxiety scale (abridged)	Anxiety	4	−0.010	Continuous, robust linear
Center for Epidemiologic Studies Depression Scale Revised (abridged) (CESD-R)	Depressive symptoms	3	−0.016	Continuous, robust linear
Self-harm	Self-harm within the last 12 months	1	N/A	Binary, logistic
Paykel suicide scale (abridged)	Suicidal ideation	4	−0.066	Continuous, robust linear

Also displayed for the multi-item measures is the change in comparative fit index (ΔCFI) that results from constraint of the factor loadings of the associated confirmatory factor analysis model to equality across countries. A ΔCFI value smaller than − 0.02 is taken as reasonable evidence of metric invariance for the scale in question

The anxiety, CESD-R and Paykel scales were shortened during the course of fieldwork, and some respondents in Cambodia, Indonesia, Thailand and Vietnam completed versions of these scales with seven, four and five items respectively. We repeated all analyses with CFA scores calculated using all items, including those removed during the fieldwork phase, as a sensitivity check. In addition, the final item in the Portuguese-language version of the CW-PWBS (“I feel positive about my future”) was mistranslated and thus all responses to this item in both the children’s and caregivers’ surveys were treated as missing.

We tested the cross-sectional association of these mental health indicators with both social connection and internet use at the time of survey completion. Participants reported whether there had ever been a lockdown in the community where they lived, and if so, whether they were able to stay in touch with friends and family who do not live with them during said lockdown. We reconfigured these responses into a single variable with three options: “no lockdown”; “lockdown, disconnected” and “lockdown, connected”. They also self-reported the frequency of their internet use at the time of the survey, which may have been outside of lockdown, on a seven-point scale from “never” to “almost all the time” (though no participants gave the response “never”, as expected given the study’s inclusion criteria).

To quantify the association between social connection or internet use and mental health, we fitted logistic and robust linear regression models as appropriate to the outcome variable type (see Table 1). We elected to use robust linear regression with Huber M-estimation [12] for continuous outcomes because of high skewness in the error distributions. We applied survey weights, as calculated by the data custodians, using the R package *survey* [13] and accounting for clustering by incorporating PSU identifiers in the specified survey design, to render the sample in each country representative of the target population. Robust linear regression models were fitted with tuning constant * k=1.345* using the R packages *MASS* [14] (unweighted) and *robsurvey* [15] (weighted). All regression models were adjusted for socioeconomic and demographic covariates alongside caregiver’s mental health status, as detailed in Table 2. Covariates were chosen to cover as many potential confounders as we could operationalise with the available data, representing potential common causes of online social connection or internet use and wellbeing outcomes. This includes variables whose potential causal relationship with the exposure and/or outcome may be indirect, e.g. indicators of socioeconomic status, and proxy variables for potential confounders, e.g. parental social attitudes as a proxy for parenting style. We also re-estimated all models separately by gender and by rural-urban status, and by each combination thereof. All models were estimated separately by country, along with a pooled model which includes all participants, with country as a covariate.

**Table 2:** Covariates included in regression models

Covariate	Number/description of items	Processing
**Included, and models were also disaggregated by these covariates**		
Gender	1	None
Rural/urban status	1 (binary)	None
**Included in all models**		
Age	1	None
How many people living in household	1	None
Single parent status of caregiver	1	None
Household income	3 (caregivers were asked whether their household had enough money for (a) food, (b) “the most important things such as clothing, school fees or medical care” and (c) “extra money for things like gifts, celebrating special occasions or taking holiday”)	Summation
Caregiver’s relationship to child	1	Recoded to a ternary variable (father, mother or other)
Caregiver’s education	1 (caregivers were asked the highest level of school or college that they attended)	Recoded to a binary variable
Caregiver’s paid employment	1 (yes or no)	None
Type of school attended	1	Recoded to a binary variable (government/public school or other)
**Included for models for internet use during the pandemic but not social connection during lockdown**		
Frequency of caregiver’s internet use	1 (on the same scale as frequency of child’s internet use)	None
Caregiver’s attitudes around sex	4 (agree or disagree with the following statements: “having sex before marriage is acceptable”; “only men, not women, should decide when to have sex”; “if someone insults a boy or man, he should defend his reputation with force if he needs to” and “a woman should tolerate violence to keep her family together”)	None (items included separately in regression models)
**Caregiver wellbeing variables (included in models with the same wellbeing variable for the child)**		
Caregiver life satisfaction	Same as in children’s survey (see Table 1)	
Caregiver CW-PWBS		
Caregiver anxiety		
Caregiver CESD-R		
Caregiver self-harm		
Caregiver Paykel suicide scale		

We used multiple imputation to handle missing data (by chained equations using the R package *mice* [16], predictive mean matching for all variables, 20 imputations, 20 iterations). Some other socioeconomic status indicators and demographic information were included in the analysis dataset as auxiliary variables in this multiple imputation, to increase the plausibility of the assumption that missing data are missing at random [17]. These were: birth order of the selected child, caregiver’s marital status, whether the child has a disability or long-standing illness or other difficulty, caregiver’s alcohol intake and caregiver’s attitude towards corporal punishment.

All data analysis was conducted in R version 4.3.2 [18] using the *targets* pipeline toolkit [19] and rendered using Quarto [20].

## Results

**Fig. 2:**
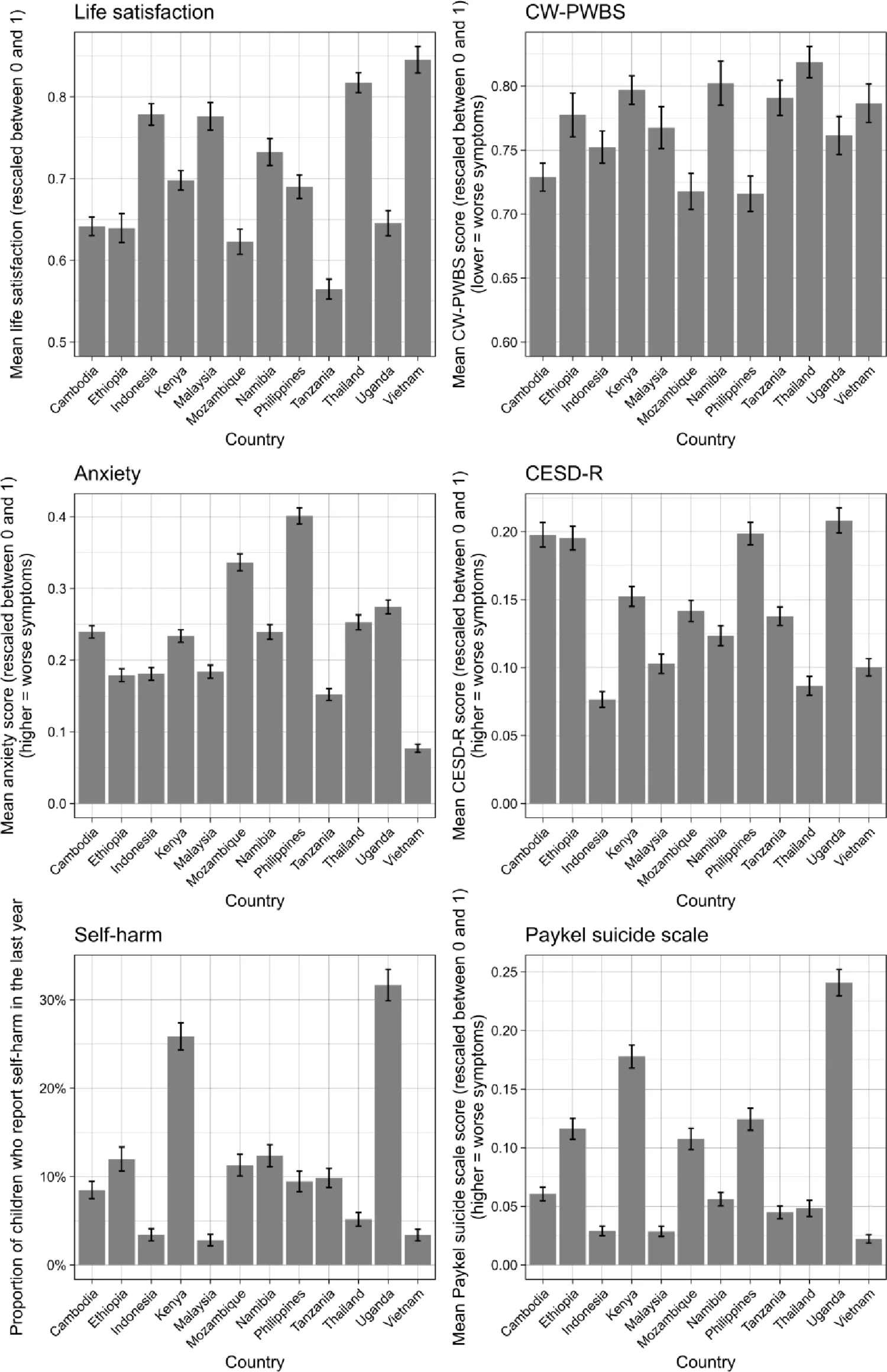
Mean scores for each of the six wellbeing scales by country (averaged across imputations). Error bars represent 95% confidence intervals

### Descriptive statistics and confirmatory factor analysis

Figure 2 shows the mean derived factor scores for each of the six wellbeing measures in each country. Of particular note are the extremely high proportions of children reporting self-harm, and extremely high mean Paykel suicide scale scores, in Kenya and Uganda. These two countries display high rates of affirmative responses across all four Paykel items, indicating that scores are unlikely to be directly comparable across countries (i.e. that an assumption of scalar invariance would not hold). For this reason, these measures should not be directly compared across countries. However, if an assumption of metric invariance were to be supported for each of the scales, then this would still allow for countries to be pooled into a single regression model, provided that the country is included in the model as a covariate. However, given the diverse range of countries included in the *Disrupting Harm* survey, we argue that the country-level results are more useful for guiding future research.

The changes in comparative fit indices (ΔCFI) resulting from constraining the factor loadings of the items in the wellbeing scales to equality across countries are displayed in Table 1. In accordance with our stated threshold for metric invariance of $\:\left|\varDelta\:\mathrm{CFI}\right|<0.02$, we found sufficient evidence for metric invariance in the modified CW-PWBS, the anxiety scale and the CESD-R. However, metric invariance is not supported for the Paykel suicide scale. Additionally, scalar invariance is not supported for any of the wellbeing measures.

In the results that follow, all regression coefficients $\:\beta\:$ are reported with respect to the mental health and internet use variables after standardisation (Z-scoring). As this is an exploratory work, we have not corrected the reported *p*-values for multiple comparisons, and the results therefore should not be taken as confirmatory evidence of any particular relationship. Because of issues with model convergence probably relating to the generally low incidence of self-harm and suicidal ideation in the sample, we were unable to estimate most of the weighted associations involving these two indicators. However, we can report the results of unweighted modelling when pooled across all countries, including country in the model as a covariate as detailed above. For the other four mental health indicators, the results from unweighted modelling were not substantially different from those of weighted modelling, but it should be noted that results relating to self-harm and suicidality reported here are not strictly generalisable to the populations of the countries involved in the study.

We were additionally unable to apply our modelling procedure in Tanzania, because there were no lockdowns in the country.

### Lockdown and social connection

**Fig. 3:**
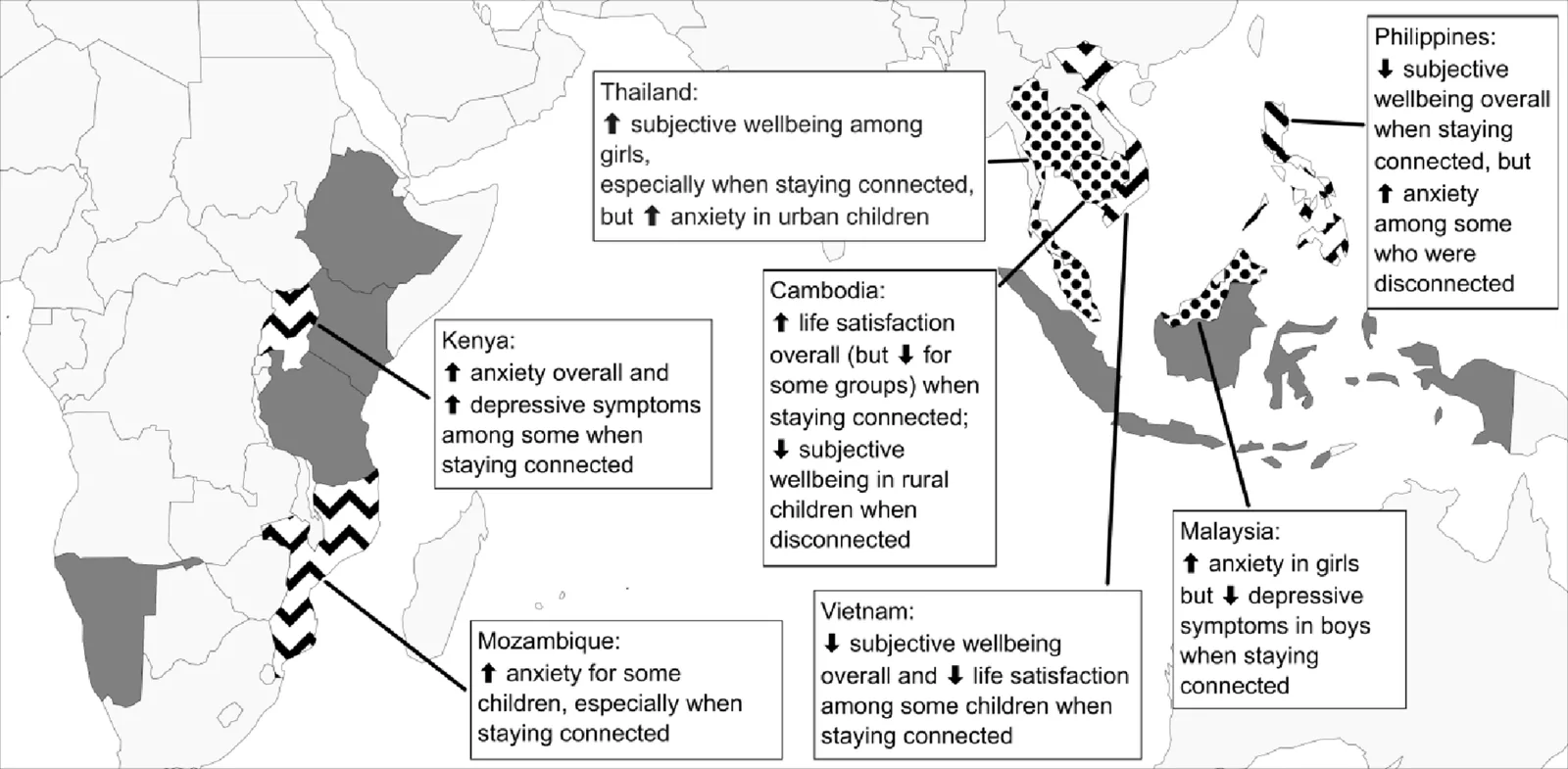
Summary of country-wise associations between lockdown (either *staying connected* to friends and family or being *disconnected*) and mental health indicators, compared to experiencing no lockdown at all. The pattern of each country represents the overall direction of the significant associations we found ((with horizontal zigzags indicating mostly adverse associations, vertical stripes indicating mostly favourable associations, spots indicating mixed findings and solid grey indicating entirely null findings), but note that in all cases most of the associations were null. Please note that the boundaries shown on the above map do not imply the expression of any opinion whatsoever on the part of the authors, their funders or UNICEF concerning the legal status of any country, territory, city or area or of its authorities, or concerning the delimitation of its frontiers or boundaries

Figure 3 shows a summary of the associations between social connection during lockdown and mental health indicators in each study country. 

#### Across countries

Considering first the models quantifying the associations between social connection during lockdown and mental health indicators across all countries, there were no significant associations applicable to the entire population. However, there was evidence for slightly higher subjective wellbeing among boys ($\beta\:=0.075\:[0.004,\:0.146],p=0.038$) and boys living in rural areas ($\beta\:=0.110\:[0.020,\:0.200],p=0.017$) who experienced a lockdown but remained connected, compared to those who experienced no lockdown. There was also evidence for increased anxiety among those girls living in rural areas who experienced a lockdown and were disconnected, compared to those who experienced no lockdown ($\beta\:=0.119\:[0.005,\:0.233],p=0.040$). In addition, a higher proportion of children living in urban areas who experienced a lockdown but stayed connected reported self-harm in the last 12 months compared to those who experienced no lockdown ($\mathrm{OR}=1.575\:[1.086,\:2.283],p=0.017$), driven by the relationship among girls living in urban areas ($\mathrm{OR}=1.791\:[1.092,\:2.939],p=0.021$). In contrast, children living in rural areas who either experienced a lockdown but remained connected ($\beta\:=-0.005\:[-0.007,-0.002],\:p<0.0005$ unweighted) or experienced no lockdown ($\beta\:=-0.004\:[-0.006,-0.001],p=0.003$ unweighted) reported very slightly lower suicidality than those who experienced a lockdown and were disconnected.

Where countries are omitted in the list that follows, it indicates that none of the regression coefficients in any of the models were statistically significant.

#### Cambodia

Children living in Cambodia who experienced a lockdown but were able to stay connected to friends and family that they did not live with reported higher life satisfaction than those who did not experience a lockdown ($\beta\:=0.198\:\left[0.027,\:0.370\right],\:p=0.024$), and this is also true among boys ($\beta\:=0.287\:\left[0.087,\:0.488\right],\:p=0.005$) and boys living in rural areas ($\beta\:=0.261\:\left[0.022,\:0.501\right],p =0.033$). However, among children living in urban areas, both having no lockdown ($\beta\:=-0.855\:\left[-1.386,\:-0.324\right],p=0.002$) and having a lockdown and remaining connected ($\beta\:=-0.692\:\left[-1.167,\:-0.217\right],p=0.004$) were associated with lower life satisfaction scores than having a lockdown and being disconnected, with only the former applying to boys living in urban areas ($\beta\:=-0.825\:[-1.397,\:-0.253], p = 0.005$) and both applying to girls living in urban areas ($\beta\:=-1.224\:\left[-2.093,\:-0.355\right],p = 0.006$ and $\beta\:=-1.383\:\left[-2.295,\:-0.471\right],p = 0.003$ respectively). There was also evidence that both the “no lockdown” and “lockdown, connected” conditions were associated with higher subjective wellbeing than the “lockdown, disconnected” condition among children living in rural areas ($\beta\:=0.249\:\left[0.017, \:0.480\right],p = 0.035$ and $\:\beta\:=0.272\:\left[0.025,\:0.519\right],p = 0.031$ respectively) and boys living in rural areas ($\beta\:=0.343\:[0.056,\:0.629],p = 0.019$ and $\:\beta\:=0.383\:[0.106,\:0.659],p = 0.007$ respectively), while the former was associated with lower subjective wellbeing among boys living in urban areas ($\beta\:=-0.621\:[-1.175,\:-0.068],p = 0.028$).  

#### Malaysia

There was evidence for increased anxiety among girls living in urban areas who experienced a lockdown and remained connected to friends and family, relative to those who did not experience a lockdown ($\beta\:=0.256\:[0.021,\:0.492],p=0.033$). However, there was also evidence for decreased depressive symptoms in boys relative to those who did not experience a lockdown ($\beta\:=-0.076\:[-0.148,\:-0.003],p=0.042$) and those who were disconnected during lockdown ($\beta\:=-0.363\:[-0.709, - 0.017], p = 0.040)$). Those boys who experienced a lockdown but were disconnected also exhibited lower levels of depressive symptoms than those who remained connected ($\beta\:=-0.439\:\left[-0.796,\:-0.081\right],\:p = 0.016$).

#### Mozambique

There was evidence for increased anxiety for those who had a lockdown but stayed connected compared to those who had no lockdown in boys ($\beta\:=0.370\:[0.056,\:0.685],p=0.021$), children living in rural areas ($\beta\:=0.352\:[0.037,\:0.666],p=0.028$) and boys living in rural areas ($\beta\:=0.452\:[0.047,\:0.857],p=0.029$). The trend towards increased anxiety also existed in children living in rural areas who had a lockdown and were disconnected, relative to those who did not experience a lockdown ($\beta\:=0.331\:[0.011,\:0.650],p=0.043$).

#### Philippines

There was evidence for reduced subjective wellbeing among those who had a lockdown but remained connected: compared to no lockdown among girls living in rural areas ($\beta\:=-0.362\:[-0.682,\:-0.042],p=0.026$) and compared to having a lockdown and being disconnected among all children ($\beta\:=-0.150\:[-0.290,\:-0.010],p=0.036$) and among girls ($\beta\:=-0.256\:[-0.459,\:-0.053],p=0.014$). By contrast, higher anxiety was reported when experiencing a lockdown but being disconnected compared to experiencing no lockdown among girls living in rural areas ($\beta\:=0.465\:[0.085,\:0.845],p=0.017$) and compared to experiencing a lockdown but remaining connected among children living in rural areas ($\beta\:=0.294\:[0.082,\:0.506],p=$ 0.007) and girls living in rural areas ($\beta\:=0.351\:[0.055,$ 0.648], *p* = 0.020).

#### Thailand

Among girls, there was evidence that experiencing a lockdown but remaining connected was associated with higher subjective wellbeing compared to experiencing no lockdown ($\beta\:=0.177\:[0.011,\:0.343],p=0.036$), while among girls living in urban areas this was true both compared to experiencing no lockdown ($\beta\:=0.242\:[0.018,\:0.465],p=0.034$) and experiencing a lockdown and being disconnected ($\beta\:=0.589\:[0.030,\:1.147],p=0.039$. There was also evidence to suggest that experiencing a lockdown but remaining connected was associated with higher anxiety in children living in urban areas ($\beta\:=-0.376\:[-0.715,\:-0.038],p = 0.029$) and girls living in urban areas ($\beta\:=-0.489\:[-0.848,\:-0.129],p = 0.008$) compared to experiencing no lockdown.

#### Uganda

There was evidence that experiencing a lockdown, regardless of whether or not social connection was maintained, was associated with higher anxiety overall (cf. disconnected: $\:\beta\:=0.323\:[0.057,\:0.589],p=0.017$; cf. connected: $\:\beta\:=0.316\:[0.049,\:0.582],\:p=0.020$). This association was particularly pronounced in boys (cf. disconnected: $\:\beta\:=0.420\:[0.086,\:0.754],\:p=0.014$; cf. connected: $\:\beta\:=0.382\:[0.077,\:0.688],p=0.014$) and boys living in rural areas (cf. disconnected: $\:\beta\:=0.506\:[0.159,\:0.854],p = 0.004$); cf. connected: $\:\beta\:=0.447\:[0.132,\:0.762],p = 0.005$). Having a lockdown and remaining connected was associated with higher depressive symptoms than having no lockdown among boys living in rural areas ($\beta\:=0.306\:[0.051,\:0.562],p=0.019$) and girls living in urban areas ($\beta\:=0.884\:[0.291,\:1.478],p=0.004$).  

#### Vietnam

Overall, having a lockdown but remaining connected was associated with lower subjective wellbeing than not having a lockdown at all ($\beta\:=-0.200\:[-0.344,\:-0.057],\:p=0.006$). Among children living in urban areas, having a lockdown but remaining connected was associated with lower life satisfaction than having a lockdown and being disconnected ($\beta\:=-0.235\:[-0.450,\:-0.020],\:p=0.032$).

### Internet use

**Fig. 4:**
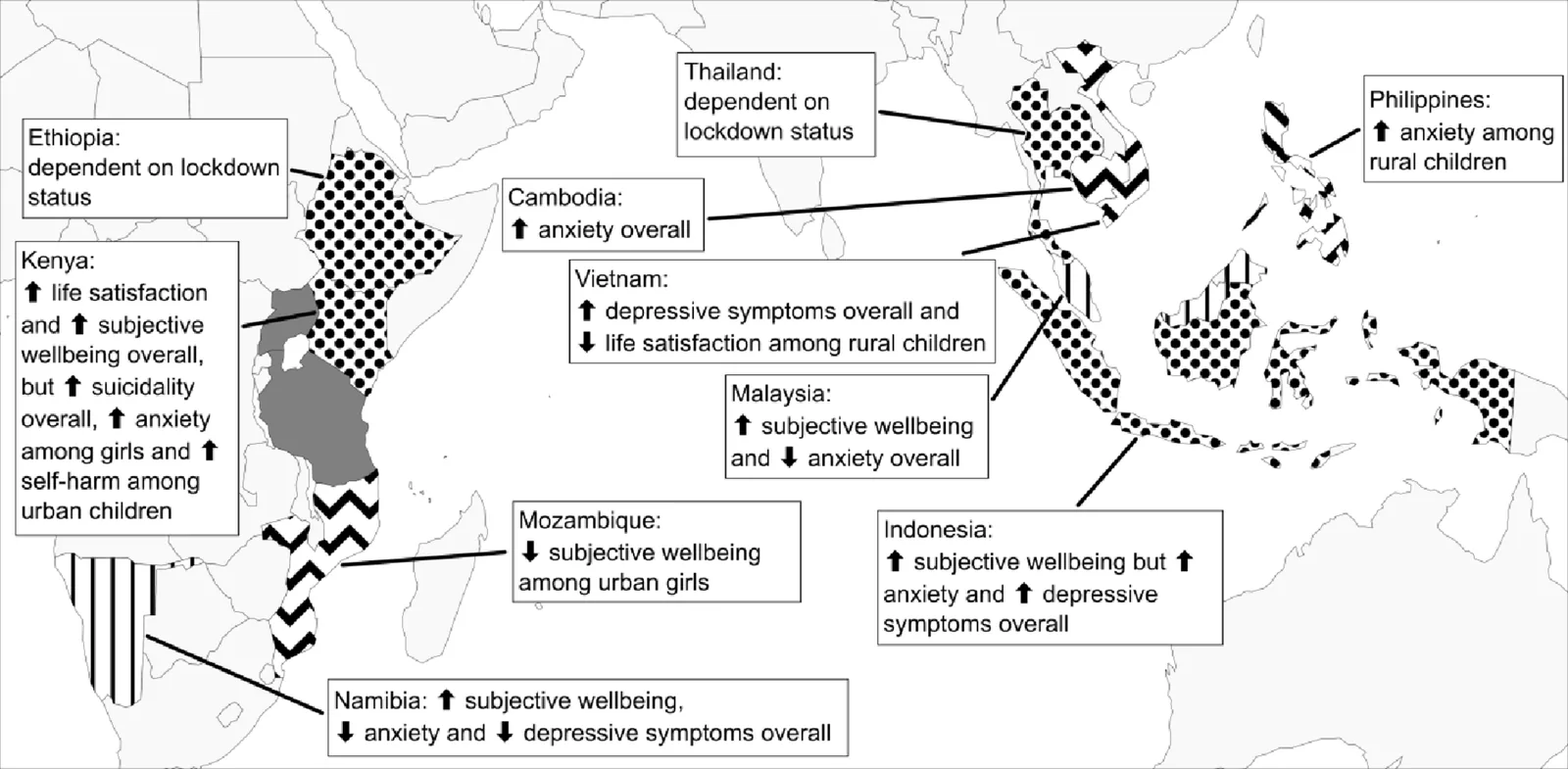
Summary of country-wise associations between internet use during the COVID-19 pandemic and mental health indicators. The pattern of each country represents the overall direction of the significant associations we found (with horizontal zigzags indicating mostly adverse associations, vertical stripes indicating mostly favourable associations, spots indicating mixed findings and solid grey indicating entirely null findings), but note that in all cases most of the associations were null. Please note that the boundaries shown on the above map do not imply the expression of any opinion whatsoever on the part of the authors, their funders or UNICEF concerning the legal status of any country, territory, city or area or of its authorities, or concerning the delimitation of its frontiers or boundaries.

Figure 4 shows a summary of the associations between internet use during the COVID-19 pandemic and mental health indicators in each study country.

#### Across countries

Among all participants, higher frequencies of internet use were associated with higher life satisfaction ($\beta\:=0.040\:[0.010,\:0.070],p=0.009$), and this was echoed among girls ($\beta\:=0.049\:[0.008,\:0.091],p = 0.019$) and children living in rural areas ($\beta\:=0.049\:\left[0.011,\:0.087\right],p = 0.011$). Internet use was also associated with higher subjective wellbeing among all participants ($\beta\:=0.063\:\left[0.032,\:0.094\right],p < 0.001$), and such an association was observed in all subgroups except boys living in urban areas. However, internet use was also associated with higher anxiety among girls living in rural areas ($\beta\:=0.061\:\left[0.009,\:0.114\right],p = 0.022$).  

Where countries are omitted in the list that follows, it indicates that none of the regression coefficients in any of the models were statistically significant.

#### Cambodia

Internet use was associated with higher anxiety among all participants ($\beta\:=0.185\:\left[0.071,\:0.298\right],\:p=0.001$), among boys ($\beta\:=0.268\:\left[0.133,\:0.404\right],\:p<\:0.001$), children living in rural areas ($\beta\:=0.213\:\left[0.111,\:0.314\right],p < 0.001$), boys living in rural areas ($\beta\:=0.284\:\left[0.145,\:0.422\right],p < 0.001$) and girls living in rural areas ($\beta\:=0.153\:\left[0.005,\:0.300\right], p = 0.042$).  

#### Ethiopia

The interaction of internet use during the pandemic with having experienced a lockdown was associated with higher life satisfaction among boys living in urban areas ($\beta\:=0.194\:\left[0.011,\:0.378\right],\:p=0.038$), but not either factor considered separately. This interaction was also associated was increased subjective wellbeing in all participants ($\beta\:=0.102\:\left[0.014,\:0.190\right],\:p=0.024$), girls ($\beta\:=0.163\:\left[0.002,\:0.323\right],\:p=0.047$), children living in rural areas ($\beta\:=0.112\:\left[0.017,\:0.207\right],\:p=0.021$) and girls living in rural areas ($\beta\:=0.284\:\left[0.137,\:0.431\right],\:p<0.001$). However, it was also associated with higher anxiety in girls living in rural areas ($\beta\:=0.112\:\left[0.006,\:0.217\right], p = 0.038$). It was also associated with lower depressive symptoms in boys ($\beta\:=-0.111\:\left[-0.180,\:-0.043\right],\:p=0.001$) and boys living in urban areas ($\beta\:=-0.156\:\left[-0.276,\:-0.036\right], p = 0.011$). In unweighted analyses, internet use was also associated with higher suicidality in girls ($\beta\:=0.059\:\left[0.005,\:0.112\right], p = 0.031$ unweighted), children living in urban areas ($\beta\:=0.082\:\left[0.014,\:0.150\right],\:p=0.018$ unweighted), boys living in urban areas ($\beta\:=0.087\:\left[0.018,\:0.156\right],\:p=0.014$ unweighted) and girls living in rural areas ($\beta\:=0.062\:\left[0.004,\:0.119\right],\:p=0.035$ unweighted). The interaction between internet use during the pandemic and having experienced a lockdown was also associated with increased suicidality in girls ($\beta\:=0.056\:\left[0.004,\:0.108\right],p = 0.035$ unweighted) and girls living in urban areas ($\beta\:=0.187\:\left[0.018,\:0.356\right],\:p=0.030$ unweighted).  

#### Indonesia

Internet use was associated with significantly higher subjective wellbeing among all participants ($\beta\:=0.084\:[0.002,\:0.165],p=0.044$). However, it was also associated with increased anxiety among all participants ($\beta\:=0.079\:\left[0.001,\:0.158\right],\:p=0.047$) and children living in urban areas ($\beta\:=0.159\:\left[0.027,\:0.291\right],\:p = 0.018$). It was also associated with increased depressive symptoms among all participants ($\beta\:=0.022\:\left[0.002,\:0.042\right],\:p = 0.028$) and children living in rural areas ($\beta\:=0.031\:[0.001, 0.062], p = 0.046$).  

#### Kenya

Internet use was associated with higher life satisfaction among all participants $\beta\:=0.085\:[0.006,\:0.165],p=0.036$), among girls living in urban areas ($\beta\:=0.195\:[0.011,\:0.380],p=0.038$) and among boys living in rural areas ($\beta\:=0.123\:[0.001,\:0.245],p = 0.048$). It was also associated with higher subjective wellbeing among all participants ($\beta\:=0.092\:\left[0.009,\:0.175\right], p = 0.031$), girls ($\beta\:=0.125\:\left[0.020,\:0.229\right],\:p=0.020$), children living in urban areas ($\beta\:=0.178\:\left[0.043,\:0.312\right],\:p=0.010$) and girls living in urban areas ($\beta\:=0.276\:\left[0.111,\:0.441\right],p = 0.001$). On the other hand, it was also associated with higher anxiety among girls ($\beta\:=0.179\:[0.060,\:0.298],p=0.003$) and girls living in rural areas ($\beta\:=0.191\:[0.049,\:0.333],p=0.008$). It was also associated with increased self-harm among children living in urban areas ($\:\mathrm{OR}=1.497\:\left[1.051,\:2.130\right], p = 0.025$) and girls living in urban areas ($\:\mathrm{OR}=1.925\:\left[1.239,\:2.989\right], p = 0.004$). It was also associated with higher suicidality among all participants ($\beta\:=0.065\:\left[0.001,\:0.130\right],\:p=0.047$), girls ($\beta\:=0.183\:\left[0.050,\:0.317\right],\:p = 0.007$) and girls living in rural areas ($\beta\:=0.206\:\left[0.057,\:0.356\right],\:p = 0.007$).

#### Malaysia

Internet use was associated with higher subjective wellbeing among all participants ($\beta\:=0.177\:[0.052,\:0.302],p=0.005$) and among children living in urban areas ($\beta\:=0.239\:[0.080,\:0.398],p=0.003$) and girls living in urban areas ($\beta\:=0.263\:[0.005,\:0.521], p = 0.046$). However, among boys living in urban areas the interaction between internet use during the pandemic and having experienced a lockdown was associated with lower subjective wellbeing ($\beta\:=-0.196\:\left[-0.388,\:-0.004\right],\:p = 0.046$). Internet use was associated with lower anxiety among all participants ($\beta\:=-0.128\:\left[-0.248,\:-0.009\right],\:p=0.035$), children living in urban areas ($\beta\:=-0.141\:-0.271,- 0.002], p = 0.046$) and girls living in urban areas ($\beta\:=-0.193\:[-0.364, - 0.022], p = 0.027$).  

#### Mozambique

Internet use was associated with lower subjective wellbeing among girls living in urban areas ($\beta\:=-0.284\:\left[-0.489,\:-0.078\right],\:p=0.007$), as was the interaction between internet use during the pandemic and having experienced a lockdown ($\beta\:=-0.163\:[-0.311, - 0.015], p = 0.031$).

#### Namibia

Internet use was associated with higher subjective wellbeing overall ($\beta\:=0.134\:[0.023,\:0.246],p = 0.018$), and among girls ($\beta\:=0.201\:\left[0.067,\:0.334\right],p = 0.003$) and children living in urban areas ($\beta\:=0.208\:\left[0.026,\:0.390\right],\:p=0.025$). Internet use was associated with lower anxiety among all participants ($\beta\:=-0.137\:\left[-0.250,\:-0.024\right],p=0.017$), boys ($\beta\:=-0.191\:\left[-0.320,\:-0.061\right],p=0.004$) and boys living in rural areas ($\beta\:=-0.212\:\left[-0.385,\:-0.039\right],p = 0.016$), however in all three cases there was a significant interaction with lockdown in the opposite direction ($\beta\:=0.107\:[0.003, 0.210], p = 0.043$; $\:\beta\:=0.159\:\left[0.036,\:0.283\right],p = 0.012$ and $\:\beta\:=0.211\:\left[0.065,\:0.358\right],p = 0.005$ respectively). Internet use was associated with lower depressive symptoms among all participants ($\beta\:=-0.158\:[-0.237,\:-0.079],p < 0.001$) and among boys ($\beta\:=-0.207\:\left[-0.335,\:-0.080\right],p = 0.001$), children living in rural areas ($\beta\:=-0.211\:\left[-0.321,\:-0.101\right],\:p<0.001$) and boys living in rural areas ($\beta\:=-0.274\:\left[-0.468,\:-0.081\right],p = 0.005$). However, the interaction between internet use during the pandemic and having experienced a lockdown was associated with increased depressive symptoms in all participants and all subgroups except girls living in rural areas. 

#### Philippines

Internet use was associated with higher anxiety among children living in rural areas ($\beta\:=0.233\:[0.034,\:0.431],p=0.022$) and boys living in rural areas ($\beta\:=0.299\:[0.078,\:0.520],p=0.008$). On the other hand, the interaction between internet use during the pandemic and having experienced a lockdown was associated with reduced depressive symptoms among girls living in rural areas ($\beta\:=-0.232\:\left[-0.403,\:-0.061\right],\:p=0.008$).

#### Thailand

The interaction between internet use during the pandemic and having experienced a lockdown was associated with reduced life satisfaction among all participants ($\beta\:=-0.092\:[-0.175,\:-0.010],p=0.028$), and children living in urban areas ($\beta\:=-0.150\:[-0.258,\:-0.041],p = 0.007$). The interaction was also associated with higher anxiety among all participants ($\beta\:=0.219\:[0.059,\:0.380],p = 0.007$), boys ($\beta\:=0.316\:[0.089,\:0.543],p=0.006$) and children living in urban areas ($\beta\:=0.439\:\left[0.210,\:0.668\right],p < 0.001$).  

#### Vietnam

Internet use was associated with lower life satisfaction among children living in rural areas ($\beta\:=-0.119\:[-0.211,\:-0.028],p=0.011$) and girls living in rural areas ($\beta\:=-0.164\:[-0.279,\:-0.050],p=0.005$). Internet use was also associated with higher depressive symptoms among all participants ($\beta\:=0.075\:\left[0.003,\:0.147\right],\:p = 0.042$), boys ($\beta\:=0.133\:\left[0.045,\:0.222\right],p = 0.003$) and boys living in urban areas ($\beta\:=0.220\:\left[0.008,\:0.432\right],p = 0.042$).  

### Sensitivity check

Our sensitivity analyses using CFA scores calculated using all questionnaire items, including those removed during fieldwork, returned very similar results.

## Discussion

In this study, we demonstrate the lack of clear cross-sectional associations between either social connection during lockdown or internet use during the pandemic and mental health indicators among adolescents in 12 countries in the Global South. While there are a number of associations that meet the threshold for significance (without correction for multiple testing), there are also a much larger number of tests that returned null findings. Nonetheless, those associations that we have highlighted in our results can serve to generate hypotheses for future research in these countries, ideally involving longitudinal study designs and more detailed questions about the nature of children’s internet use, outside of the online child sexual exploitation focus of the *Disrupting Harm* survey.

When considering the mental health associations of social connection during lockdown, some themes emerge. Across all countries, we saw that experiencing a lockdown but remaining connected with family and friends whom respondents did not live with was associated with higher subjective wellbeing than experiencing no lockdown among boys and among boys living in rural areas, though the size of this association is very small. However, these overarching results mask the fact that the results are highly different among countries, and the findings are not straightforward to summarise. There were notably few associations between a lack of social connection and depressive symptoms as measured by the abridged CESD-R, but anxiety was elevated among at least some of those who experienced a lockdown in several countries.

We noted a general association between a high frequency of internet use and increased life satisfaction and subjective wellbeing, though again this effect was quite small, and again this obscures heterogeneity among countries. For example, in Cambodia, more frequent internet use was generally associated with higher anxiety, in Indonesia it was associated with increased depressive symptoms, and in Vietnam it was associated with lower life satisfaction among girls and children living in rural areas. In some cases, internet use was associated with very different mental health indications between genders and between rural and children living in urban areas, demonstrating that considering the different uses of the internet by different children will be a crucial component of future research. There are likely also additional factors not considered here that could modify the relationship between children’s internet use and mental health, such as sexual orientation [21]. Though we faced difficulties in obtaining model convergence to estimate the associations between self-harm or suicidal ideation and internet use, the results that we were able to obtain are also highly heterogeneous.

We argue that our results are broadly inconsistent with the notion that there is a strong and uniform association between online social connection and mental health symptoms during COVID-19–related lockdowns in the countries involved in this study. If, as hypothesised by some (e.g. [6]), use of digital technologies to maintain social connection during lockdowns acted as a protective factor for mental health, we would expect to see positive associations between both maintaining social connection during lockdown and internet use during the pandemic and wellbeing indicators. If, on the other hand, increased reliance on digital connection was broadly harmful, we would expect to identify more consistent negative associations in this study. Our findings point to a much more complex picture, understanding the nuances of which requires significant further theoretical developments in conjunction with more detailed future research studies.

This study has a number of strengths, particularly relating to its sample. Nationally representative samples are not common features of surveys in the Global South, for example because of the high cost of recruiting such a sample given gaps in infrastructure and other challenges. However, there is enormous added value from doing so, as we were able to examine wellbeing even in rural populations that are often not reached by studies of mental health in the Global South. In addition, we were able to apply sampling weights that render our findings generalisable to the populations (aged 12–17) of each country in turn. This is the first step in increasing the representation of countries in the Global South in research about how digital technologies are related to child wellbeing. Also, the *Disrupting Harm* survey employs a wide range of wellbeing measures, which allows us to start to build a more nuanced understanding of the ways in which digital technologies may be related to different mental health outcomes.

However, there are also many limitations, and our findings should not be treated as authoritative and certainly not as suggesting any form of causality, given the cross-sectional design. Few of the associations we identified would be statistically significant when corrected for multiple comparisons. Given the small effect sizes we would expect when investigating the relationship between an exposure as broad as internet use, the sample of 1,000 children per country and the rarity of phenomena such as self-harm and suicidality, the study was also hindered by statistical power limitations. The lack of scalar invariance (and metric invariance, in the case of the Paykel suicide scale) in the wellbeing measures employed here mean we must caution against drawing comparisons across countries on the basis of these data. We have additionally been unable to take into consideration the differences in activities that children take part in online, which are likely to be important when considering the relationship between internet use and mental health symptoms. It is also important to consider that the country-level results mask significant ethnocultural and demographic diversity within countries, beyond the delineations of gender and urbanicity that we were able to capture. Finally, we were unable to fully account for the complex survey design in the imputation process because of computational limitations of the multiple imputation by chained equations algorithm, which introduces the potential for bias as the imputation and substantive analysis models cannot strictly be considered compatible [22].

Extensive further research is needed to investigate the relationship between both online social connection and internet use and young people’s mental health in each of the countries involved in this study, and to support the development of more detailed theories of how digital technologies are related to child wellbeing. This research should employ mixed-methods approaches and longitudinal designs, in order firstly to identify specific online behaviours that may support or undermine mental wellbeing, secondly to be able to gain a sense of the temporal directionality of associations identified and thirdly to more fully understand the experiences of ethnoculturally and demographically diverse communities. There is also an area of opportunity surrounding the development and use of cross-culturally validated measures of wellbeing [23]. Our inability to establish scalar invariance for the mental health measures used in this study demonstrates that there may be differences in how these measures are perceived in different cultural contexts. This would tally with existing research spotlighting some of the barriers to mental health treatment presented by different conceptualisations of wellbeing among cultures [24]. It is possible that this could affect the interpretation of some of our findings and those that follow from it in subsequent research.

Many concerns have been raised in both mainstream media and academic circles about the possible effects of digital technologies on mental health. In spite of the implications that this would have for global mental health, there is a chronic lack of diversity in samples used to investigate these concerns [9]. As such, marked developments in digital wellbeing research outside the Global North are needed to facilitate equitable care in the mental health sphere globally. Therefore, we believe that the directions for future research that we have indicated should be treated as urgent priorities by funders and policymakers.

## Supplementary Information

Below is the link to the electronic supplementary material.

Supplementary Material 1

## Data Availability

The supplementary data for this article, including the code used for analysis, are available at [https://doi.org/10.17605/OSF.IO/342QY](https:/doi.org/10.17605/OSF.IO/342QY). The original data used are controlled by UNICEF Innocenti – Global Office of Research and Foresight and cannot be shared by the authors.
