# Supplementary material for: Children’s social connections, internet usage and mental health during the COVID-19 pandemic in the Global South: evidence from Disrupting Harm
Source: Soc Psychiatry Psychiatr Epidemiol. 2026 Aug 4;61(12):2669–82. doi: 10.1007/s00127-026-03101-8 (PMC13727930; doi:10.1007/s00127-026-03101-8)
Supplement: Supplementary file 1 [file 127_2026_3101_MOESM1_ESM.pdf]

Supporting information for

Children's social connections, internet usage and mental health during the COVID-19 pandemic in the Global South: evidence from Disrupting Harm

Thomas E. Metherell*, Sebastian Kurten, Sakshi Ghai, Daniel Kardefelt-Winther, George B. Ploubidis, Darío Moreno-Agostino & Amy Orben

* Correspondence should be addressed to Tom Metherell
Email: thomas.metherell.22@ucl.ac.uk

Country	Ethical approval
**Kenya**	National Commission for Science, Technology and Innovation
**Uganda**	Makerere University School of Public Health, the Uganda National Council of Science and Technology
**Cambodia**	Cambodia National Council for Children and the Ministry of Interior
**Mozambique**	Ministry of Health, National Committee on Bioethics for Health at a national level
**Malaysia**	Medical Research and Ethics Committee
**Indonesia**	Health Research Ethics Committee, National Institute of Health Research and Development
**Namibia**	Ministry of Health and Social Services Ethical Review Board
**Vietnam**	Ministry of Labour, Invalids and Social Affairs (MOLISA)
**Philippines**	Philippine Social Science Council Ethical Review Board (SSERB)
**Ethiopia**	Ethiopian Society of Sociologists, Social Workers and Anthropologists
**Tanzania**	Medical Research, the National Bureau of Statistics, and the President's Office, Regional Administration and Local Government. Permits from the Tanzania Commission for Science and Technology. Additional approvals were sought from the Zanzibar Health Research Institute.
**Thailand**	Reviewed by a special panel of reviewers from Institute of Human Rights and Peace Studies at Mahidol University given that no formal government process or requirement for social research to be ethically reviewed.

Table S1. Details of the ethical approval processes of UNICEF Innocenti – Global Office of Research and Foresight's research components in each study country. In addition, UNICEF Innocenti – Global Office of Research and Foresight and ECPAT International's research components were reviewed and approved by the Health Media Lab Institutional Review Board.
